# Supplementary material for: EphB2-Targeting Monoclonal Antibodies Exerted Antitumor Activities in Triple-Negative Breast Cancer and Lung Mesothelioma Xenograft Models
Source: Int J Mol Sci. 2025 Aug 27;26(17):8302. doi: 10.3390/ijms26178302 (PMC12428355; doi:10.3390/ijms26178302)
Supplement: Supplementary file 1 [file ijms-26-08302-s001.zip › ijms-3818595-supplementary.pdf]

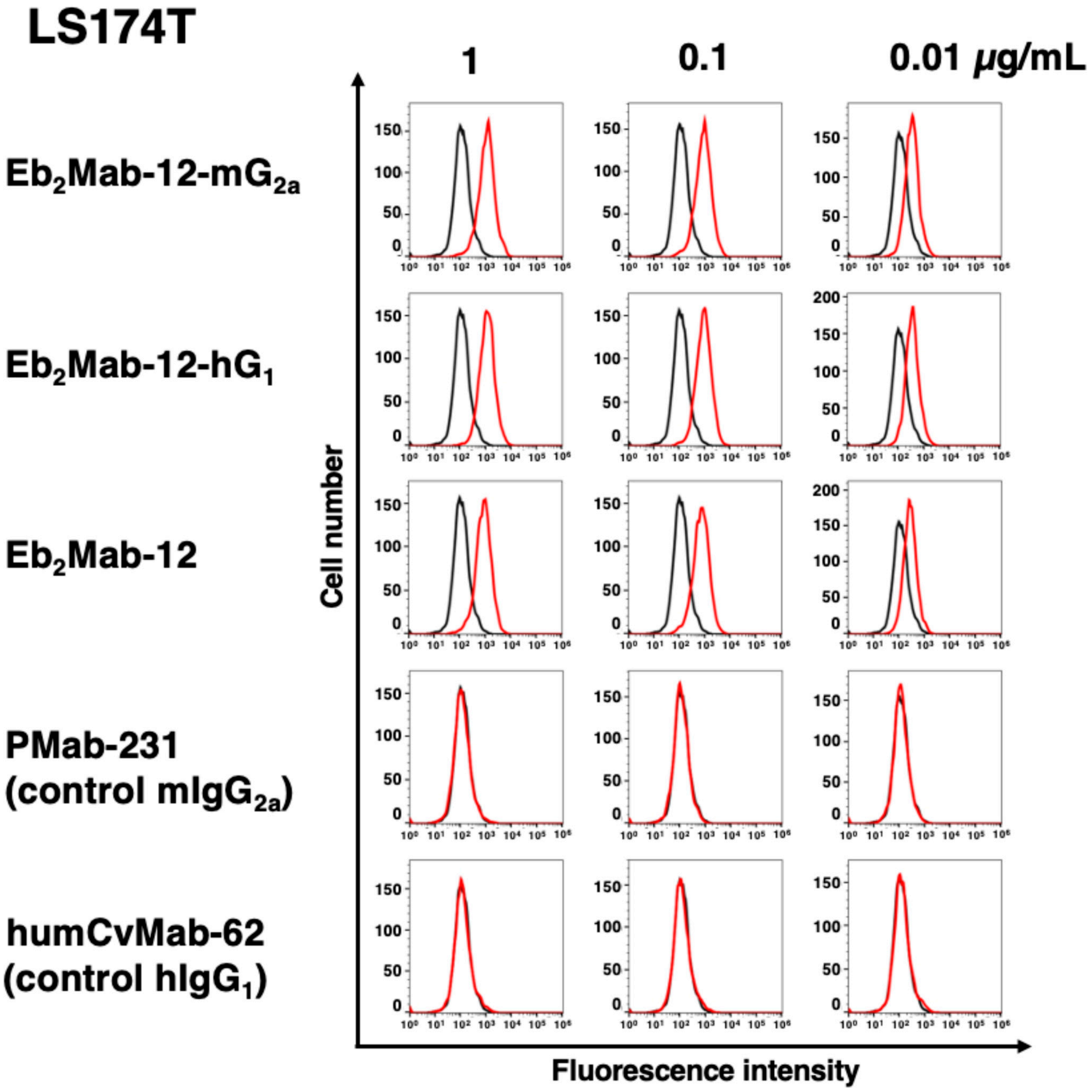

Supplementary Figure S1. Flow cytometry analysis of Eb2Mab-12, Eb2Mab-12-mG2a, and Eb2Mab-12-hG1 to colorectal cancer (LS174T). Cells were treated with 0.01, 0.1, and 1 μg/mL of indicated mAbs. Then, the cells were treated with Alexa Fluor 488-conjugated anti-mouse IgG or FITC-conjugated anti-human IgG. Fluorescence data were analyzed using the SA3800 Cell Analyzer.

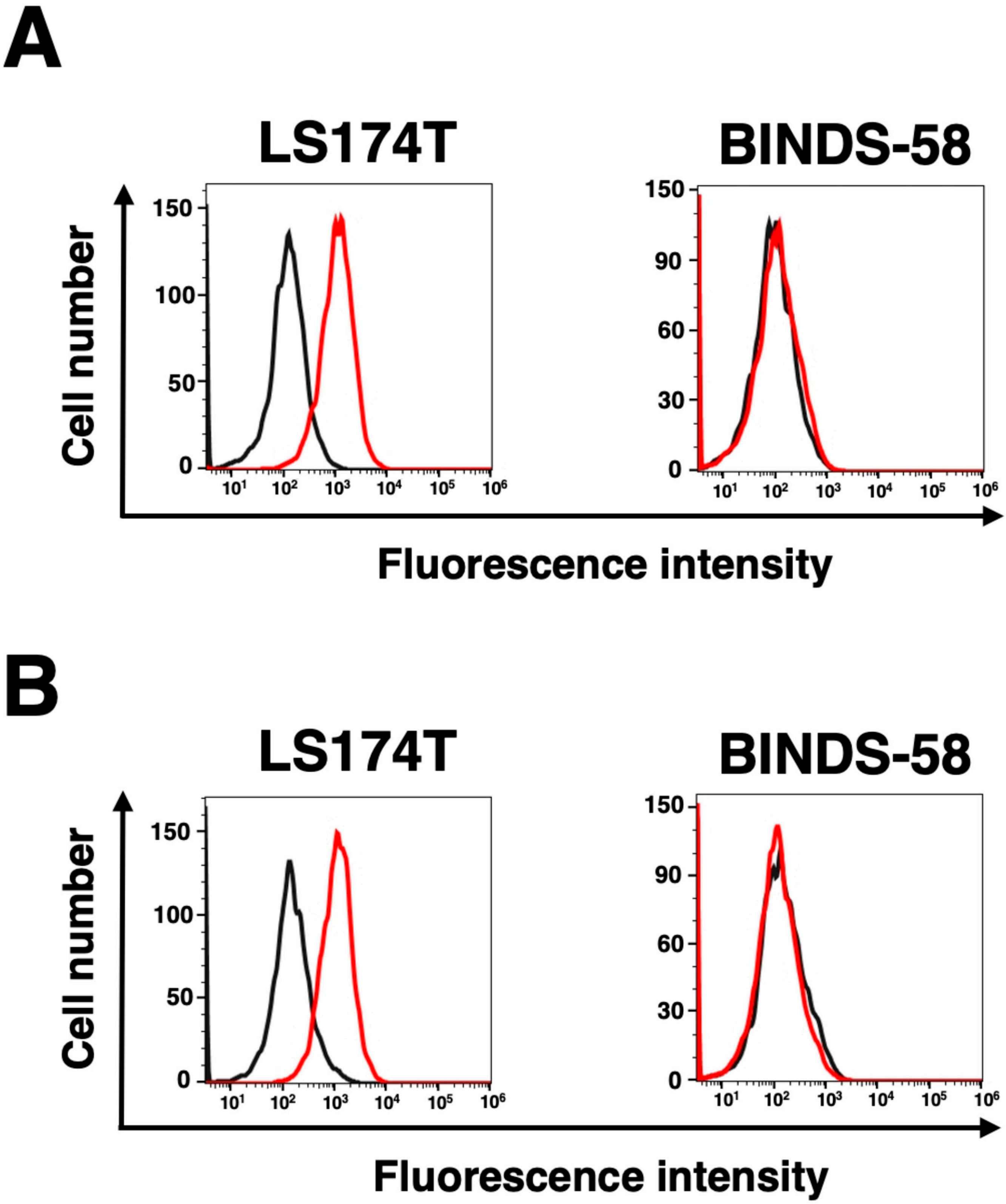

Supplementary Figure S2. Flow cytometry analysis of Eb2Mab-12-mG2a and Eb2Mab-12-hG1 to LS174T and EphB2-knockout LS174T (BINDS-58). (**A**) LS174T and BINDS-58 were treated with 0.1 µg/mL of Eb2Mab-12-mG2a (red) or control mouse IgG2a (mIgG2a, black). (**B**) LS174T and BINDS-58 were treated with 0.1 µg/mL of Eb2Mab-12-hG1 (red) or control human IgG1 (hIgG1, black). Then, the cells were treated with Alexa Fluor 488-conjugated anti-mouse IgG or FITC-conjugated anti-human IgG. Fluorescence data were analyzed using the SA3800 Cell Analyzer.

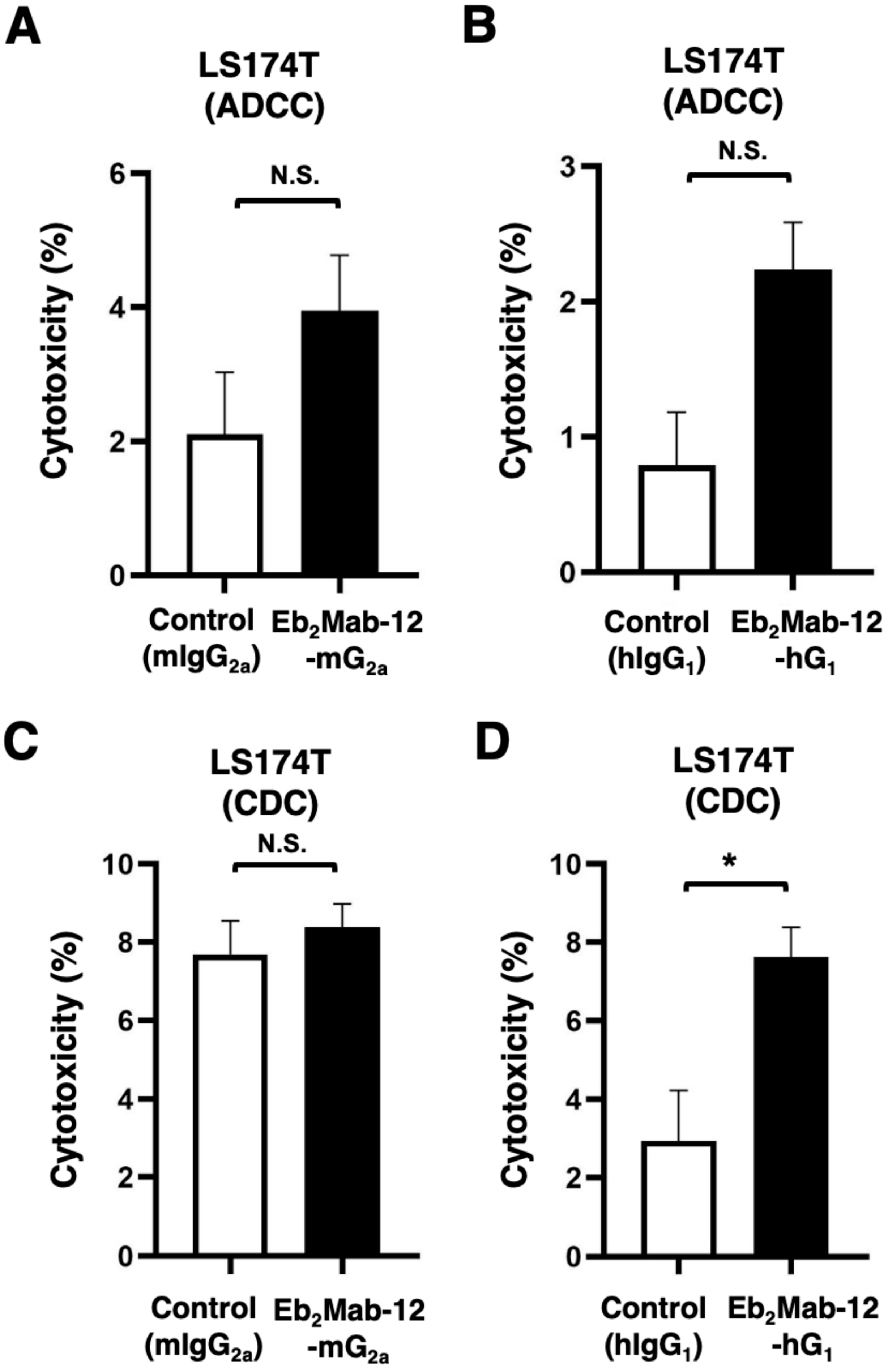
A
LS174T
(ADCC)
N.S.
Cytotoxicity (%)
0
2
4
6
Control
(mIgG2a)
Eb2Mab-12
-mG2a
B
LS174T
(ADCC)
N.S.
Cytotoxicity (%)
0
1
2
3
Control
(hIgG1)
Eb2Mab-12
-hG1
C
LS174T
(CDC)
N.S.
Cytotoxicity (%)
0
2
4
6
8
10
Control
(mIgG2a)
Eb2Mab-12
-mG2a
D
LS174T
(CDC)
*
Cytotoxicity (%)
0
2
4
6
8
10
Control
(hIgG1)
Eb2Mab-12
-hG1

Supplementary Figure S3. ADCC and CDC by Eb_2Mab-12-mG_{2a} and Eb_2Mab-12-hG_1 against LS174T. (**A**) ADCC induced by Eb_2Mab 12-mG_{2a} or control mouse IgG_{2a} ($mIgG_{2a}$) against LS174T. (**B**) ADCC induced by Eb_2Mab-12-hG_1 or control human IgG_1 ($hIgG_1$) against LS174T. (**C**) CDC induced by Eb_2Mab-12-mG_{2a} or $mIgG_{2a}$ against LS174T. (**D**) CDC induced by Eb_2Mab-12-hG_1 or $hIgG_1$ against LS174T. Values are shown as mean ± SEM. Asterisks indicate statistical significance (* $p < 0.05$; Two- tailed unpaired t test). N.S., not significant.

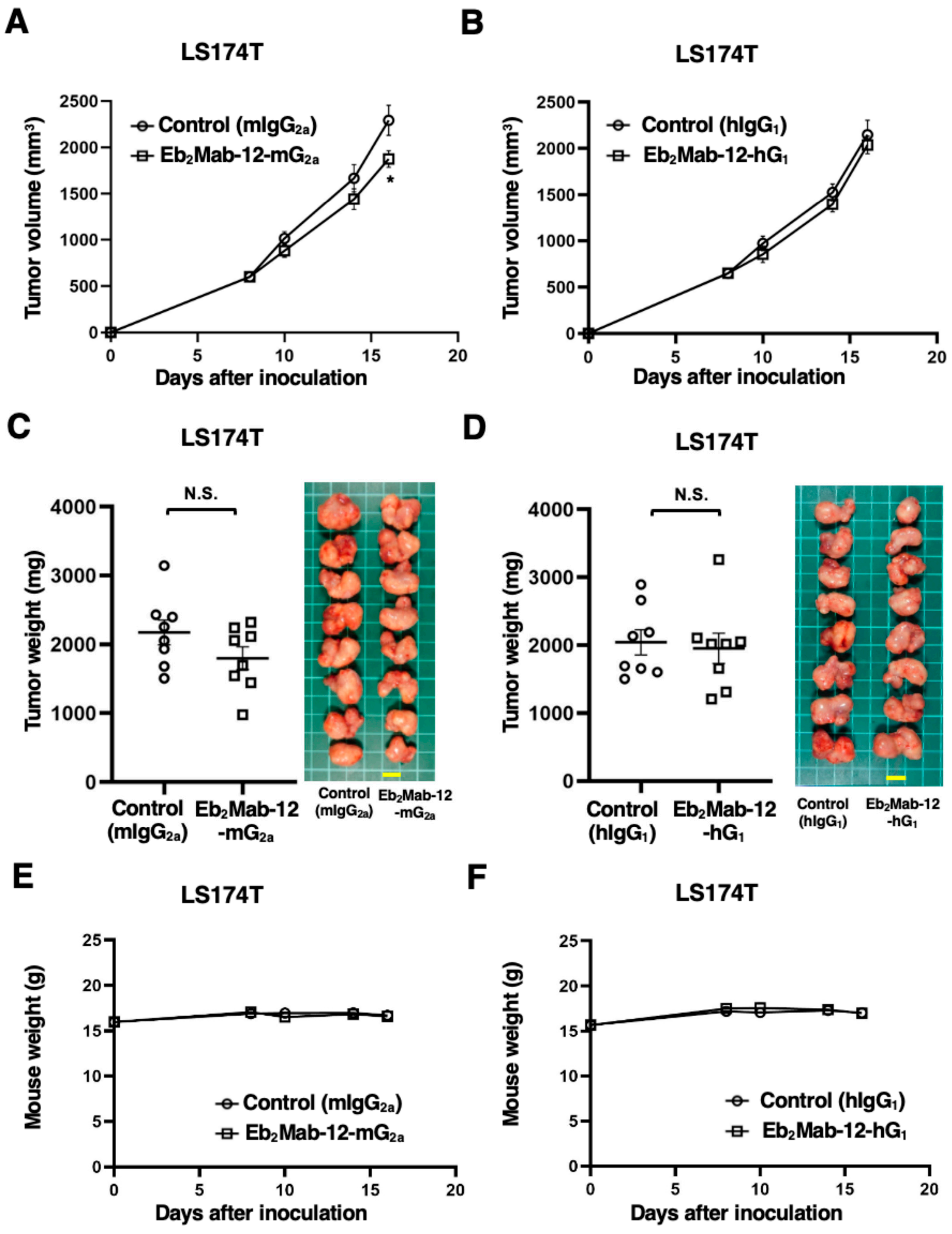

Supplementary Figure S4. Antitumor activity of Eb2Mab-12-mG2a and Eb2Mab-12-hG1 against LS174T xenograft. LS174T were subcutaneously injected into BALB/c nude mice (day 0). (**A**) In total, 100 μg of Eb2Mab-12-mG2a or control mouse IgG2a (mIgG2a) were intraperitoneally injected into each mouse on day 8. Additional antibodies were injected on day 14. (**B**) In total, 100 μg of Eb2Mab-12-

hG_1 or control human IgG_1 ($hIgG_1$) were intraperitoneally injected into each mouse on day 8. Additional antibodies were injected on day 14. The tumor volume is represented as the mean ± SEM. * $p < 0.05$ (ANOVA with Sidak's multiple comparisons test). (**C,D**) The mice treated with above-mentioned mAbs were euthanized on day 16. The LS174T xenograft weights were measured. Values are presented as the mean ± SEM. N.S., not significant. (**E,F**) Body weights of LS174T xenograft-bearing mice treated with above-mentioned mAbs. There is no statistical difference.
